# Tuning catalytic performance of platinum single atoms by choosing the shape of cerium dioxide supports†

**DOI:** 10.1039/d4cy00484a

**Published:** 2024-08-14

**Authors:** Petrus C. M. Laan, Martijn J. Mekkering, Felix J. de Zwart, Alessandro Troglia, Roland Bliem, Kai Zhao, Norbert J. Geels, Bas de Bruin, Gadi Rothenberg, Joost N. H. Reek, Ning Yan

**Affiliations:** a Van't Hoff Institute for Molecular Sciences, University of Amsterdam Science Park 904 1098XH Amsterdam The Netherlands g.rothenberg@uva.nl; b Advanced Research Center for Nanolithography (ARCNL) Science Park 106 1098XG Amsterdam The Netherlands; c Key Laboratory of Artificial Micro- and Nano-Structures of Ministry of Education, School of Physics and Technology, Wuhan University Wuhan 430072 China ning.yan@whu.edu.cn

## Abstract

The local coordination environment of single atom catalysts (SACs) often determines their catalytic performance. To understand these metal–support interactions, we prepared Pt SACs on cerium dioxide (CeO_2_) cubes, octahedra and rods, with well-structured exposed crystal facets. The CeO_2_ crystals were characterized by SEM, TEM, pXRD, and N_2_ sorption, confirming the shape-selective synthesis, identical bulk structure, and variations in specific surface area, respectively. EPR, XPS, TEM and XANES measurements showed differences in the oxygen vacancy density following the trend rods > octahedra > cubes. AC-HAADF-STEM, XPS and CO-DRIFTS measurements confirmed the presence of only single Pt^2+^ sites, with different surface platinum surface concentrations. We then compared the performance of the three catalysts in ammonia borane hydrolysis. Precise monitoring of reaction kinetics between 30–80 °C gave Arrhenius plots with hundreds of data points. All plots showed a clear inflection point, the temperature of which (rods > octahedra > cubes) correlates to the energy barrier of ammonia borane diffusion to the Pt sites. These activity differences reflect variations in the – facet dependent – degree of stabilization of intermediates by surface oxygen lone pairs and surface–metal binding strength. Our results show how choosing the right macroscopic support shape can give control over single atom catalysed reactions on the microscopic scale.

## Abstract 2

Shape up: the macroscopic shape of cerium oxide supports changes the catalytic performance of Pt single atom catalysts (SACs) in ammonia borane hydrolysis.

## Graphical abstract

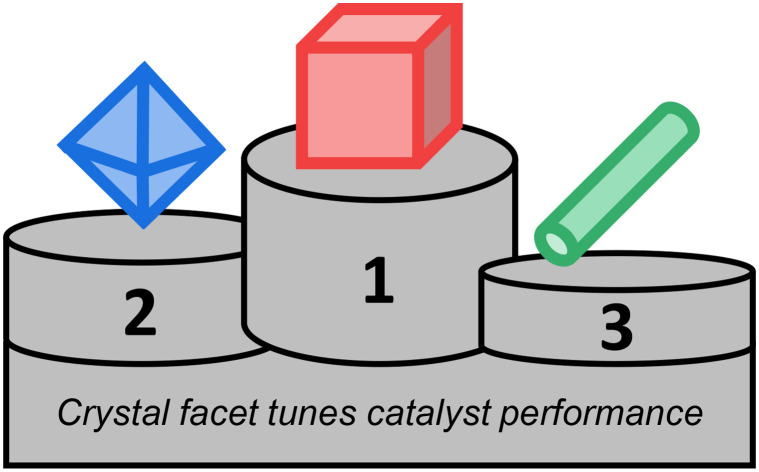

## Introduction

Minimizing the ecological footprint of the chemical industry relies on the development of new and better catalysts.^1^ Most of these materials are noble-metal nanoparticles supported on inexpensive metal oxides. To maximize noble-metal usage, single atom catalysts (SACs) are being developed extensively.^2,3^ These catalysts consist of atomically dispersed noble-metal atoms which are bound by multiple support atoms. The physico-chemical properties of the support and the electronic metal–support interactions (EMSIs) play an important role in determining the catalytic performance of SACs – just like a ligand does for molecular catalysts.^4^

Controlling these EMSIs is crucial. Cerium dioxide (CeO_2_) is known for having strong EMSIs promoting high metal dispersity as well as tailoring catalytic performances for nanoparticle-based catalysts.^5,6^ These strong interactions are mainly controlled by surface oxygen vacancies which differ per crystal facet. CeO_2_ crystals can be synthesized as cubes, octahedra or rods, and each shape features a unique terminal facet: (100), (111) and (110), respectively. Controlling the shape of the CeO_2_ support is thus a straightforward way for tailoring these EMSIs and has led to developments in sensing, solar cells and catalysis.^7^ There are indeed various reports in the literature showing the shape (facet) effects of ceria supported catalysts. Yet, understanding such effects remains challenging for typical heterogeneous catalysts that consists out of supported transition metal nanoparticles. On one hand, only the metal atoms close to the support are subject to strong EMSIs whereas reactions eventually occur across the entire surface of the nanoparticle. On the other hand, the control of facet effects is often accompanied by the change of other physico-chemical parameters of ceria (*e.g.*, defect density, surface area) which will affect the catalytic performance as well. Therefore, disentangling the real facet effects from various influential factors remains ambiguous.^8,9^

In this context, we prepared platinum SACs on CeO_2_ cubes, octahedra and rods with distinct surface facets and studied their catalytic performance in the solvent phase, an environment which is rarely probed on the study of EMSIs using SACs.^10–13^ Using aberration corrected scanning transmission electron microscopy (AC-STEM) and diffuse reflectance infrared Fourier transform spectroscopy of adsorbed CO (CO-DRIFTS), we examined the surface defect structure of ceria on various facets and confirmed the atomic distribution of Pt. A deeper understanding of the defect chemistry was obtained by investigating X-ray absorption near edge structure (XANES) and electron paramagnetic resonance spectroscopy (EPR). In the ammonia borane hydrolysis carried out at various temperatures, all catalysts showed a kink in their Arrhenius plots at different temperatures which reflects the surface travel of ammonia borane to the active sites. Our results show that the shape of the support particles influences the stability of Pt sites and reaction intermediates, and thereby the catalytic activity.

## Results and discussion

### Synthesis and characterization of CeO_2_ cubes, octahedra and rods

**Fig. 1:**
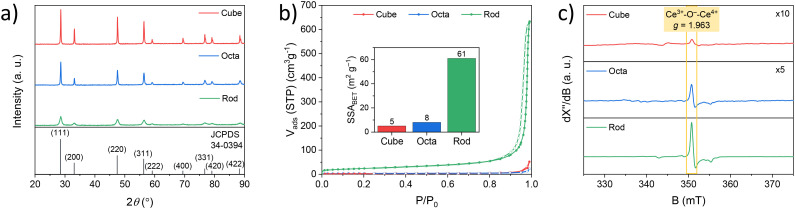
Characterization of CeO_2_ cubes, octahedra and rods. (a) Powder X-ray diffraction (pXRD) patterns, (b) N_2_ sorption isotherms at 77 K (inset: corresponding specific surface area (SSA) determined using the Brunauer–Emmett–Teller (BET) method), and (c) CW X-band electron paramagnetic resonance (EPR) spectra (*T* = 293 K and *v* = 9.643 GHz).

To study how the surface structure of CeO_2_ is related to the catalytic performance of supported platinum SACs, first CeO_2_ cubes, octahedra and rods were synthesized according to literature procedures.^14–16^ Shape selective synthesis was confirmed by scanning electron microscopy (SEM) measurements (Fig. S1†). The phase structure of the samples was analysed by powder X-ray diffraction (Fig. 1a). Their diffraction patterns were similar and could be indexed as the face-centred cubic (fcc) structure of CeO_2_ (JCPDS 34-0394) confirming that the bulk of the material is identical. Yet the peak width and intensity both decreased (rod > octa > cube), indicating an increase in crystallite size and periodicity, which is in line with the particle sizes observed by SEM imaging. We then ran nitrogen sorption experiments to study the accessible surface area and porosity of the samples (Fig. 1b and S2–S4†). These showed only type II sorption isotherms, with minimal hysteresis which is typical for nonporous adsorbents.^17^ The calculated specific surface area (SSA) as determined by the Brunauer–Emmett–Teller (BET) method are similar for cubes (5 m^2^ g^−1^) and octahedra (8 m^2^ g^−1^) but is significantly larger for the rod-shaped particles (61 m^2^ g^−1^).

Most importantly, we probed the density of oxygen vacancies by CW X-band electron paramagnetic resonance (EPR) spectroscopy at room temperature (Fig. 1c).^18,19^ The typical Ce^3+^–O^−^–Ce^4+^ (*g* = 1.963) defect was observed in all samples as indicated in yellow.^20–23^ What differs among the samples is the signal intensity, which follows the order rod > octa > cube. While exact quantification remains difficult, this shows that the density of oxygen defects is directly correlated to the terminal crystal facet type.^24^

### Synthesis and characterization of platinum single atoms on CeO_2_ cubes, octahedra and rods

With the differently shaped CeO_2_ supports at hand, we synthesised platinum SACs on CeO_2_ cubes (Pt_1_@cube), octahedra (Pt_1_@octa) and rods (Pt_1_@rod), using wet impregnation of hexachloroplatinic acid (see ESI† for details). Inductively coupled plasma optical emission spectrometry (ICP-OES) confirmed that all samples had a similar bulk platinum content (Table S1†). We then studied the oxygen vacancy densities of the three catalysts by Ce and O X-ray photoelectron spectroscopy (XPS, Fig. S5 and S6 and Table S2†). These show the same trend (rod > octa > cube) as determined by EPR (Fig. 1c) before impregnation, validating that impregnating Pt did not alter the oxygen vacancy density trend in the surface region. This same trend was obtained from X-ray absorption near edge structure (XANES) studies on the K-edge of O and the M_4,5_-edge of Ce too (Fig. S7 and S8†).^25^

**Fig. 2:**
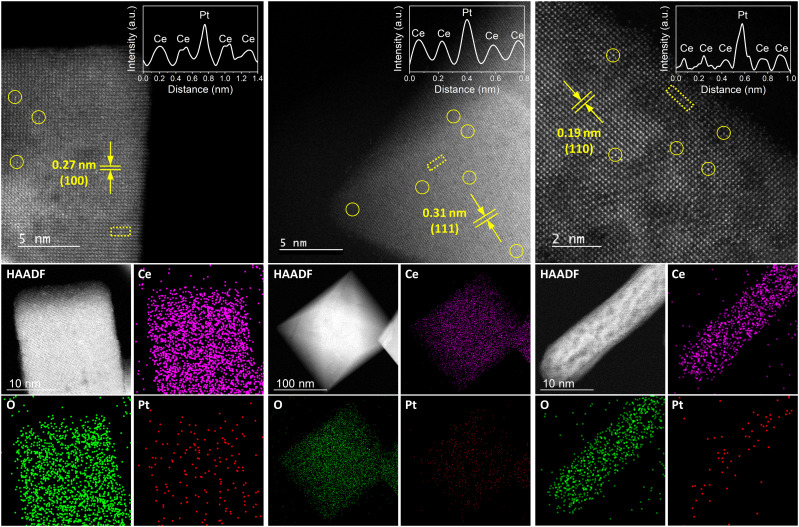
Structural characterization of Pt_1_@cube, Pt_1_@octa and Pt_1_@rod. Aberration corrected HAADF-STEM imaging (top) and energy-dispersive X-ray (EDX) spectroscopy (bottom) of Pt_1_@cube (left), Pt_1_@octa (middle) and Pt_1_@rod (right). Spatially isolated Pt atoms are indicated by the yellow circles. The areas for intensity profiling are indicated by the yellow squares and represent two peaks with different intensity assigned to Ce and Pt, according to their atomic number. The intensity profiles are shown at the top right corner of the corresponding AC-HAADF-STEM image.

Next, we ran aberration-corrected HAADF-STEM measurements to study the structure and dispersion of Pt on the three supports (Fig. 2). These show the known interplanar spacings for CeO_2_ cubes (100), octahedra (111) and rods (110) of 0.27, 0.31 and 0.19 nm, respectively, confirming the expected terminal facets.^14–16^ Moreover, they show the atomic dispersion of Pt on the surfaces of all three catalysts without the formation of any clusters or nanoparticles.

**Fig. 3:**
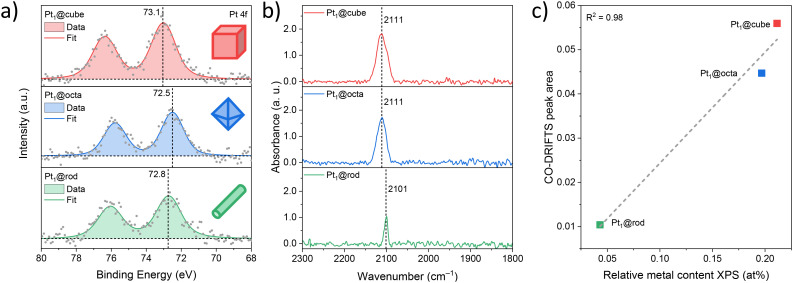
Characterization of Pt_1_@cube, Pt_1_@octa and Pt_1_@rod. (a) High-resolution X-ray photoelectron spectroscopy (XPS) spectra of the Pt 4f region, (b) CO diffuse reflectance infrared Fourier-transform spectroscopy (CO-DRIFTS) spectra, and (c) linear fit between the XPS-based relative metal content and CO-DRIFTS peak area.

We then studied the oxidation state and surface concentration of platinum using X-ray photoelectron spectroscopy (XPS) and diffuse reflectance infrared Fourier transform spectroscopy of adsorbed CO (CO-DRIFTS, Fig. 3). The XP spectra of the Pt 4f core levels were all dominated by one component with minor core level shifts among the samples (<0.6 eV), indicating a single and highly similar type of platinum species close to a 2+ oxidation state for all catalysts (Fig. 3a).^26,27^ The CO-DRIFTS was used for evaluating the electronic structure and dispersion of Pt (Fig. 3b).^28^ All samples show a symmetrical vibration band around 2105 cm^−1^ which is characteristic of linear bound CO on isolated Pt^2+^ atoms.^29–31^ The lack of an additional band around 2030 cm^−1^ indicates the absence of linearly bound CO on Pt^0^, confirming that all of the platinum on the catalyst surface is Pt^2+^, in agreement with the XPS data. More importantly, the absence of additional bands around 1950 cm^−1^ (CO adsorbed on the interface between Pt clusters and support) and 1860 cm^−1^ (bridge bound CO on two Pt atoms) strongly suggests that Pt was atomically dispersed on all the samples and not agglomerated into larger clusters, in line with AC-HAADF-STEM-EDX data. The identical platinum oxidation state of all three samples (Pt^2+^) allows us to study purely the facet effect.^32^ Next, we determined the amount of Pt atoms present at the surface by XPS (Table 1).

**Table 1:** Surface compositions of Pt_1_@cube, Pt_1_@octa and Pt_1_@rod

Catalyst	Surface Pt content^a^ (at%)	SSA_BET_^b^ (m^2^ g^−1^)	Average Pt_1_–Pt_1_ distance^a^^,^^b^ (nm)
Pt_1_@cube	0.21	5	1.4
Pt_1_@octa	0.20	8	1.7
Pt_1_@rod	0.04	61	11.0

a Based on XPS.

b Based on N_2_ physisorption (see ESI† for details and calculations).

The relative metal content was proportional to their corresponding CO-DRIFTS peak areas (Fig. S9–S11†). These amounts do depend on the crystals' shape, following the trend cube > octa > rod.^33^ This cross-correlation between two independent measurement techniques is important, as it confirms that both techniques provide information solely on the surface Pt species. Based on these values and the respective SSAs of the supports, we estimated the average distances between two Pt atoms (Table 1). The Pt_1_–Pt_1_ distance is significantly shorter for Pt_1_@cube and Pt_1_@octa compared to Pt_1_@rod. However, it is unlikely that their catalytic behavior will be influenced by their neighbouring SACs – this is generally observed at metal-to-metal distances below 1.2 nm only.^34^ This is further supported by the fact that we only see Pt^2+^ species at the surface in all samples (*vide supra*). Thus, the catalysts differ in only two aspects: their density of oxygen defects and their density of Pt^2+^ sites.

### Catalytic hydrolysis of ammonia borane with Pt_1_@cube, Pt_1_@octa and Pt_1_@rod

Following the characterization of the catalysts, we assessed their performance in the hydrolysis of ammonia borane in water at ambient pressure between 30 and 80 °C (eqn (1)). We chose this benchmark reaction because it requires a catalyst to proceed, and the kinetics can be accurately monitored by quantifying the hydrogen evolution. Yet this reaction has more than pure academic interest – ammonia borane is a potential hydrogen storage material due to its high hydrogen content of 19.6 wt%.^35^

1 NH_3_BH_3_ + 4H_2_O → NH_4_^+^ + B(OH)_4_^−^ + 3H_2_

**Fig. 4:**
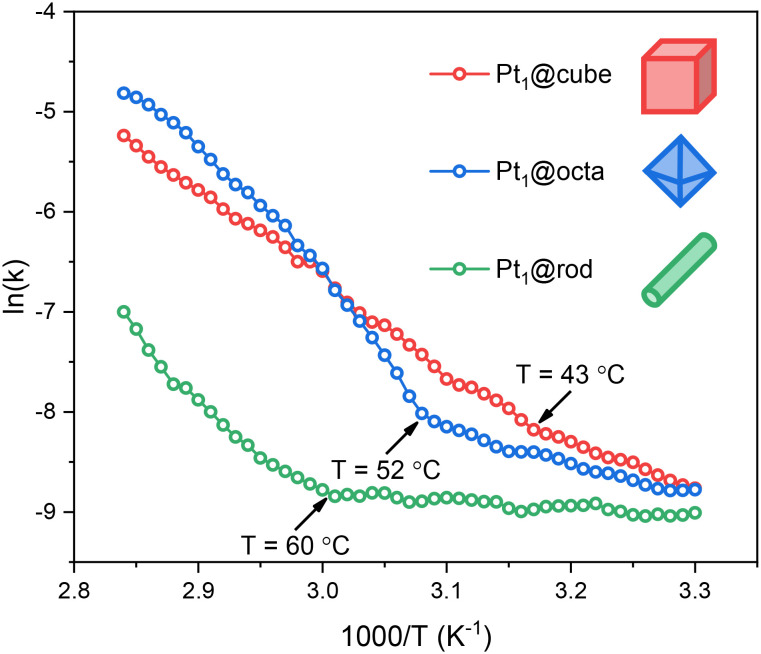
Arrhenius plots of Pt_1_@cube, Pt_1_@octa and Pt_1_@rod in the hydrolysis of ammonia borane. Each data point represents a window average of 30 individual measurements. All experiments were performed in duplo and the data shown here represent the averaged values.

The rate of the reaction over this temperature range was determined by quantifying the amount of hydrogen evolved in a single experiment by applying a temperature ramp of 2 °C using a home-built bubble counter.^36^ This simple and inexpensive setup measures gas evolution with volume steps of *ca.* 10 μL, generating highly precise Arrhenius plots based on hundreds of data points. Therefore, we can study even subtle physico-chemical changes of the catalysts. The supports themselves were not active in the reaction (Table S3†) and rate data is corrected for the amount of Pt present at the surface (see Arrhenius plots in Fig. 4).

All three catalysts show low- and high-temperature regions separated by a clear inflection point. Before this kink, the activation energies and pre-exponential factors are relatively low, indicating moderate rates. Thereafter the rates increase significantly (Fig. S12†). Such a kink in the Arrhenius plot often reflects a change in reaction mechanism. However, when we ran additional isothermal experiments at temperatures before and after the kink we observed similar kinetic profiles, only at different rates (Fig. S13 and S14†). These reaction profiles are characteristic of a zero-order rate in ammonia borane, indicating that O–H bond cleavage in water is the rate-determining step.^37^ Interestingly, this kink is not observed for ceria-supported Pt nanoparticles (*cf.* Fig. S15†), affirming that our catalysts are SACs rather than nanoparticles.

Based on this, we maintain that water activation is the rate-determining step between 30–80 °C. The kinks in the Arrhenius plots are not caused by a change in mechanism, but rather by temperature-dependent surface diffusion of ammonia borane to the Pt active sites. Because water is both reactant and solvent, the active sites are nearly always occupied by water molecules (the [H_2_O] : [NH_3_BH_3_] ratio is *ca.* 550 : 1). Yet as both substrates are required for a successful reaction, the rate depends on the availability of ammonia borane at the active site, and therefore on the travel of ammonia borane to this site.^37^ This surface travel depends on the respective surface–adsorbate binding constants and on the distance to be travelled (*i.e.* the active site density and distribution). Surface oxygen defects are known to stabilize such adsorbates, increasing the energy barrier for surface travel.^38^ Given the trends in oxygen defect density (rod > octa > cube) and active site density (rod < octa < cube), the energy barrier for ammonia borane traveling to the active site should follow the trend rod > octa > cube. Indeed, we see this in our Arrhenius plots: with increasing temperatures, the cubes show the kink first (43 °C) followed by the octahedra (52 °C) and the rods (60 °C).

To further test this hypothesis, we increased the NH_3_BH_3_ : catalyst ratio to see if this would indeed enhance the ammonia borane diffusion to the Pt sites. We did this by running a non-isothermal ammonia borane experiment at 50% of the catalyst loading while keeping the ammonia borane concentration identical and using Pt_1_@octa as an example. We see that the kink temperature decreased from 52 °C to 38 °C (Fig. S16†). This indicates that ammonia borane diffusion to the Pt sites has a lower barrier at higher relative substrate concentrations which is in line with the observed trend for the different facets.

Besides the fact that the Arrhenius plots showed kinks, the slopes also differ significantly (Fig. 4). This means that the activation energies for ammonia borane hydrolysis on Pt SACs depend on the facet they are supported on. This is partially caused by the differences in support–ammonia borane binding (*vide supra*), yet there are more parameters governing catalyst performance which are facet dependent. Firstly, Pt SACs bind stronger to CeO_2_ supports with more oxygen defects.^10,11^ Thus, based on the EPR, XPS and XANES data, the surface–metal atom binding strength should be rod > octa > cube (Fig. 1c and S6†). This means that Pt atoms supported on cubes are available for catalysis at lower temperatures compared to octahedra and rods (Fig. 4 and S12†). Secondly, reaction intermediates can be stabilized by surface oxygen atom lone pairs on ceria based catalysts.^39–41^ Reactions are therefore faster when the active site is surrounded by oxygen atoms rather than by surface defects – the most common surface defect is an oxygen vacancy in the surface lattice.^7^ This further supports the rod < octa < cube activity trend.

The recyclability of the Pt_1_@cube catalyst in ammonia borane hydrolysis was also tested at 65 °C for three consecutive runs (Fig. S17†). The maximum conversion and initial rate decrease in each run, likely due to catalyst poisoning by metaborate salts (commonly seen in borohydride hydrolysis^42^). Deactivation due to Pt leaching is less likely,^43^ as one would expect activity of the leached species. This was affirmed by SEM-imaging showing plate-like aggregates on the surface of the catalyst material (Fig. S18,†*cf.* with Fig. S1a† that shows the pristine cube-shaped crystals).

## Conclusions

Understanding the real facet effects for supported metal catalysts is challenging. By controlling the shape of CeO_2_ supports, we regulated the exposed crystal facet to tailor its properties. Using kinetic data at various temperatures, we show that the catalytic performance of Pt SACs differs significantly depending on the terminal crystal facet on which they are supported. We correlate this to the structure of these surfaces and show that the oxygen defects on each facet play a decisive role through different interactions with the supported metal atoms, substrates, and reaction intermediates. Overall, we show how nanostructuring of the support can be used to tailor the catalytic performance of ceria-based SACs. In the true Sabatier spirit, the optimal support should have (i) only isolated oxygen vacancies to stabilize the SAC with just enough energy, preventing agglomeration and leaching while keeping the metal atom available for catalysis and (ii) a surface–adsorbate binding constant high enough to facilitate adsorption yet not too high that it prevents surface travel to the active site.

## Experimental section

### General considerations

All reactions were carried out in air at room temperature unless noted otherwise. To prevent cross-contamination of trace metals, all glassware used were single use scintillation vials or glassware which was cleaned with *aqua regia* (nitric acid and hydrochloric acid in a molar ratio of 1 : 3) before use. All water used was demineralized water which was deionized by the Milli-Q technique and has a resistivity greater than 18.2 MΩ cm at room temperature and a total organic carbon content lower than 3 ppb. All reagents were purchased from commercial suppliers and used without further purification unless mentioned otherwise. Specifically, ammonia borane (technical grade, 90%), polycrystalline CeO_2_ (nano powder, <25 nm particle size), Ce(NO_3_)_3_·6H_2_O (99%), NaOH (99.99%) and H_2_PtCl_6_·6H_2_O (trace metal basis) were obtained from Sigma Aldrich, Na_3_PO_4_·12H_2_O (98–100%) was obtained from Merck and KBr (99%) was obtained from VWR International. CeO_2_ cubes, octahedra and rods were synthesized according to the procedures of Over,^14^ Xing,^15^ Hensen^16^ and their co-workers, respectively.

### Instrumentation and characterization methods

Dark field scanning electron microscopy (SEM) was performed on a FEI Verios 460 (using 5 kV electrons) equipped with an Oxford Xmax 80 mm^2^ silicon drift detector. Samples were dispersed in ethanol (±0.01 mg in 1 mL) by sonication for 1 hour before drop casting on lacey carbon center-marked grids/Cu (200 mesh grid, Ted Pella Inc.).

N_2_ adsorption–desorption isotherms were measured on a Thermo Scientific Surfer instrument at 77 K, using vacuum-dried samples. More specific, around 100 mg of each sample was dried at 100 °C for 16 h on a Belprep-vacIII prior to analysis. The specific surface area was determined based on the adsorption branch and the BET analysis was performed according to the Rouquerol consistency criteria (Fig. S2–S4†).^44,45^

Powder X-ray diffraction (pXRD) patterns were obtained with a Rigaku MiniFlex II diffractometer (Tokyo, Japan) using Ni-filtered CuKα radiation (*λ* = 1.541874 Å) at 30 kV and 15 mA. For each measurement, the sample was ground and loaded on a monocrystalline silicon sample holder with an 8 mm wide and 0.2 mm deep cavity. The powdered sample was pressed firmly in the cavity to make a uniform flat sample area. Residual sample outside the sample cavity was removed to minimize background scattering. Diffraction patterns were collected between the 2*θ* range of 20° and 90° using a rotation speed of 2° min^−1^, a step size of 0.05° and 1 s dwell time.

CW X-band electron paramagnetic resonance (EPR) spectra of the samples were measured in EPR quartz tubes on a Bruker EMX-plus CW X-band spectrometer at room temperature. The effective *g* values were defined as the magnetic field strength at the maximum microwave absorption according to eqn (S1) in which *g*_eff_ is the effective *g*-value, *h* is Planck's constant being 4.135 × 10^−15^ eV s, *v* is the microwave frequency of the spectrometer being 9.643 GHz, *μ*_B_ is the Bohr magneton being 5.788 × 10^−5^ eV T^−1^ and *B* is the applied magnetic field at the maximum microwave absorption maximum in T (1 Gauss = 1 × 10^−4^ T).S1
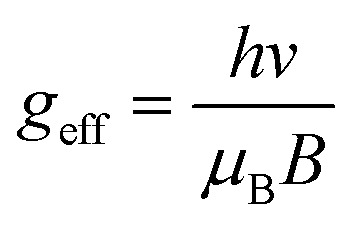


Metal loadings were determined based on inductively coupled plasma optical emission spectrometry (ICP-OES) analysis performed by the Mikroanalytisches Laboratorium Kolbe, Oberhausen, Germany. Samples were prepared using microwave digestion and then analysed with a Spectro Arcos analyzer of Spectro capable of maintaining a standard error of ±1.5 ppm.

Aberration-corrected high angle annular dark-field scanning transmission electron microscopy (AC-HAADF-STEM) measurements, elemental mappings, and energy-dispersive X-ray (EDX) spectroscopy measurements were taken on a JEOL JEM-ARM300F2 GRAND ARM™ 2 instrument coupled with a high-angle annular dark field (HAADF) detector and an energy-dispersive X-ray spectroscopy detector. The set-up was operated at 300 kV and delivers a spatial resolution of ≤60 pm in both TEM and STEM resulting in atomic-resolution imaging of the samples. Intensity profiles and interplanar spacings were determined using the Gwyddion 2.61 software package.

Ce M_4,5_-edge and O K-edge X-ray absorption near edge structure (XANES) spectra were obtained at the X-ray Magnetic Circular Dichroism (XMCD) beamline of Hefei Light Source (HLS). After baseline correction, the Ce M_4,5_-edge data was fitted by standard Gaussian curves using peak fitting in Origin 2018.

X-ray photoelectron spectroscopy (XPS) was performed in ultra-high vacuum (base pressure below 2 × 10^−9^ mbar) using a Scienta Omicron HiPP-3 analyzer with a 1 mm entrance slit operated in Swift Acceleration mode and a monochromatic Al Kα source. Survey spectra were acquired at a pass energy of 500 eV and the high-resolution spectra were acquired at pass energies of 100 eV (Pt_1_@cube and Pt_1_@octa) or 300 eV (Pt_1_@rod). Prior to data processing, the binding energies were calibrated using that of adventitious carbon (C 1s at 284.8 eV). The Ce 3d and O 1s regions are fitted using peak positions and ratios from literature.^46^ XPS peak fitting was performed using KolXPD (Kolibrik), employing a (ranged) Shirley background and Voigt functions for the individual components.

Diffuse reflectance infrared Fourier-transform spectroscopy (DRIFTS) measurements were performed on a Nicolet iS50 FTIR (Thermo Fisher, United States) spectrometer equipped with a MCTA detector and a KBr beam splitter. The diffuse reflection accessory used was a Praying Mantis™ (DRP, Harrick, United States) in combination with a high temperature reaction chamber with ZnSe windows (HVC-DRM-5, Harrick, United States) and a temperature control unit (ATK-024-4, Harrick, United States). Typically, ∼50 mg KBr was put in the reaction chamber which was topped with 10 mg of finely grounded sample. This was placed in the *in situ* cell and pre-treated at 200 °C under vacuum (typically 5 × 10^−4^ mbar, Pfeiffer HiCube) for 30 minutes followed by a pure O_2_ (99.999%) flow for 30 minutes to remove any other gases, adsorbed water and other impurities. After cooling to room temperature, the cell was vacuumed again, and a background spectrum was collected. Then, the cell was saturated with CO (99.9%). After 1 min, the cell was vacuumized (5 × 10^−4^ mbar) and the spectra were recorded in the range between 4000 and 800 cm^−1^ with a spectral resolution of 4.0 cm^−1^ until no changes between the collected spectra were visible. Each spectrum was recorded by averaging 32 scans. The peak areas were determined by peak fitting using a standard Gaussian curve using Origin 2018 (Fig. S8–S10†).

### Catalyst preparation: Pt_1_@cube/octa/rod

Hexachloroplatinic acid hexahydrate (H_2_PtCl_6_·6H_2_O) was impregnated on the different CeO_2_ supports (CeO_2_ cubes, CeO_2_ octahedra or CeO_2_ rods) by a conventional wet impregnation method. CeO_2_ (200 mg) was finely dispersed water (10 mL) by ultrasonication in a 10 mL round bottom flask for one hour and the suspension was stirred vigorously afterwards. Then, 80 μL of an aqueous 12.7 mM chloroplatinate precursor solution was added dropwise under vigorous stirring at room temperature. After 1 hour, the solvent was allowed to evaporate gently using a rotary evaporator (150 rpm at 40 °C: 30 min at 55 mbar, 30 min at 50 mbar, 4 h at 45 mbar). The CeO_2_ support completely covered the flask to minimize platinum deposition on glass and sintering there-on. The obtained dry white powder was further dried under vacuum (1 mbar at room temperature) for sixteen hours. They were subsequently calcined in static air at 500 °C for 2 h with a ramp rate of 5 °C min^−1^ and named Pt_1_@cube, Pt_1_@octa and Pt_1_@rod, respectively.

### Catalyst preparation: Pt NPs@polycrystalline CeO_2_

The Pt NPs@polycrystalline CeO_2_ were synthesized using a 800 μL of an aqueous 12.7 mM chloroplatinate precursor solution, aiming at a 1 wt% Pt loading. The rest of the synthetic procedure is equal to that of Pt_1_@cube/octa/rod.

### Kinetic studies of ammonia borane hydrolysis

Reaction kinetics of ammonia borane hydrolysis were studied using a homebuilt bubble counter of which the design^36^ and data processing^37^ is described in detail elsewhere. Briefly, after a stirring catalyst solution was at the desired temperature, the reactor was closed off and an aqueous ammonia borane solution was directly injected into the reaction mixture causing a small volume displacement. This, and any further gas evolution caused by the hydrolysis of ammonia borane forming hydrogen gas was detected by analysing bubble formation from a hexadecane medium. Bubbles were detected with the aid of a laser and translated into an evolved volume of gas. Corrections for gas expansion of the head space of the reactor and increased vapor pressure of the used solvent at elevated temperatures were made and corrected for in all experiments. Reaction rates were corrected for the fraction of platinum atoms present at the surface region of the catalysts based on XPS.

For non-isothermal ammonia borane hydrolysis, a screwcap vial (10.0 mL) was charged with a stirring bean (8.0 × 3.0 mm), catalyst (7.0 mg) and water (8.0 mL). The catalyst was homogeneously suspended with the aid of an ultrasonic bath for ten minutes at room temperature. The screwcap vial was mounted on the reactor head. A freshly prepared ammonia borane solution (0.4 mL, 2.0 M) was loaded into syringe (1.0 mL) equipped with a glass capillary (∅ = 0.32 mm, *l* = 15 cm). The capillary was inserted through one of the syringe ports into the reaction mixture and purged with nitrogen (5.0 mL min^−1^) while the reaction mixture was cooled down to 5.0 °C by an external ice bath. When this temperature was reached, the reactor was placed in the heating mantle under stirring and the other three remaining syringe ports were closed off. After five seconds, the ammonia borane solution was injected and a ramp of 2.0 °C min^−1^ was initiated achieving a controlled temperature increase between 30 and 80 °C. The sample was held at 80.0 °C until no gas production was observed anymore.

For isothermal ammonia borane hydrolysis, a screwcap vial (10.0 mL) was charged with a stirring bean (8.0 × 3.0 mm), catalyst (7.0 mg) and water (8.0 mL). The catalyst was homogeneously suspended with the aid of an ultrasonic bath for ten minutes at room temperature. The screwcap vial was mounted on the reactor head. A freshly prepared ammonia borane solution (0.4 mL, 0.2 M) was loaded into syringe (1.0 mL) equipped with a glass capillary (∅ = 0.32 mm, *l* = 15 cm). The capillary was inserted through one of the syringe ports into the reaction mixture and purged with nitrogen (5.0 mL min^−1^) while pre-heating the reaction mixture to the desired temperature under stirring by the use of a heating mantle. When stabilized at the desired temperature, the other three remaining syringe ports were closed off and the ammonia borane solution was injected. Gas production was monitored until reaction completion keeping the temperature of the reaction mixture at the desired temperature.

For the recyclability tests, the reaction mixture was centrifuged at 4000 rpm for 10 min after reaction completion in its original screwcap vial, giving a white residue and a colorless supernatant. The supernatant was removed with a needle and replaced by fresh water to a total volume of 8.0 mL. Thereafter, the purified specimen was resuspended by ultrasonication for 15 min. This washing procedure was repeated three times before a subsequent catalytic reaction was started. After the last run, the supernatant was removed, and the remaining white powder was further dried under vacuum (5 mbar at 50 °C) for 16 h. The remaining catalyst was weighed, confirming that there were no losses during the washing procedures.

## Data availability

As far as possible, all of the data is presented in the paper and in the ESI.† All other data on which the paper and the ESI† is based is available from the authors upon reasonable request.

## Author contributions

P. C. M. L., G. R., J. N. H. R. and N. Y. conceived and guided the project. P. C. M. L. performed the catalyst characterization and reactivity studies. M. J. M. performed the catalyst synthesis and CO-DRIFTS measurements. F. J. d. Z. and B. d. B. performed the EPR measurements and data analysis. A. T. and R. B. performed the XPS measurements and data analysis. K. Z. performed TEM and XANES measurements. N. J. G. performed the nitrogen adsorption measurements. P. C. M. L. and G. R. wrote the manuscript with input from all authors.

## Conflicts of interest

There are no conflicts to declare.

## Supplementary Material

CY-014-D4CY00484A-s001
