# Supplementary material for: Tuning catalytic performance of platinum single atoms by choosing the shape of cerium dioxide supports
Source: Catal Sci Technol. 2024 Aug 14;14(19):5662–70. doi: 10.1039/d4cy00484a (PMC11322700; doi:10.1039/d4cy00484a)
Supplement: CY-014-D4CY00484A-s001 [file CY-014-D4CY00484A-s001.pdf]

Supplementary Information (SI) for Catalysis Science & Technology.
This journal is © The Royal Society of Chemistry 2024

Table of contents

1. Scanning electron microscopy 1
2. Nitrogen physisorption 2
3. Additional calculations surface content platinum 5
4. X-ray photoelectron spectroscopy 6
5. X-ray absorption spectroscopy 9
6. CO diffuse reflectance infrared Fourier transform spectroscopy 10
7. Kinetic studies of ammonia borane hydrolysis 12

1. **Scanning electron microscopy**

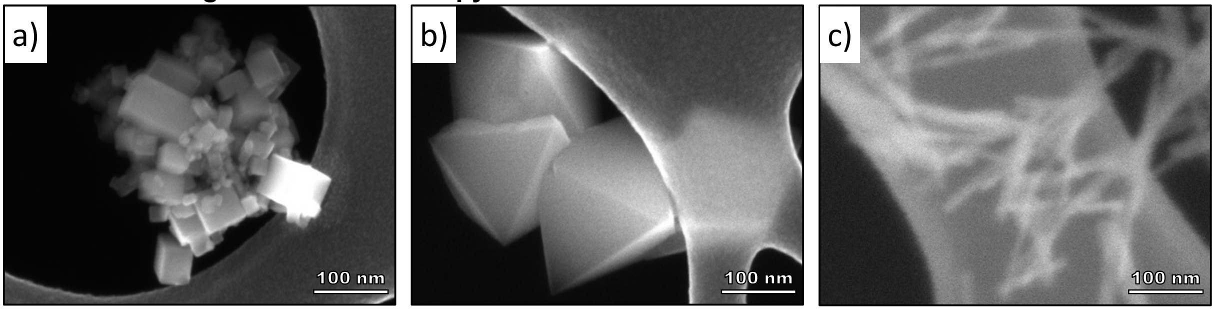

Figure S1. Scanning electron microscope (SEM) images of CeO_2 (a) cubes, (b) octahedra and (c) rods.

2. Nitrogen physisorption

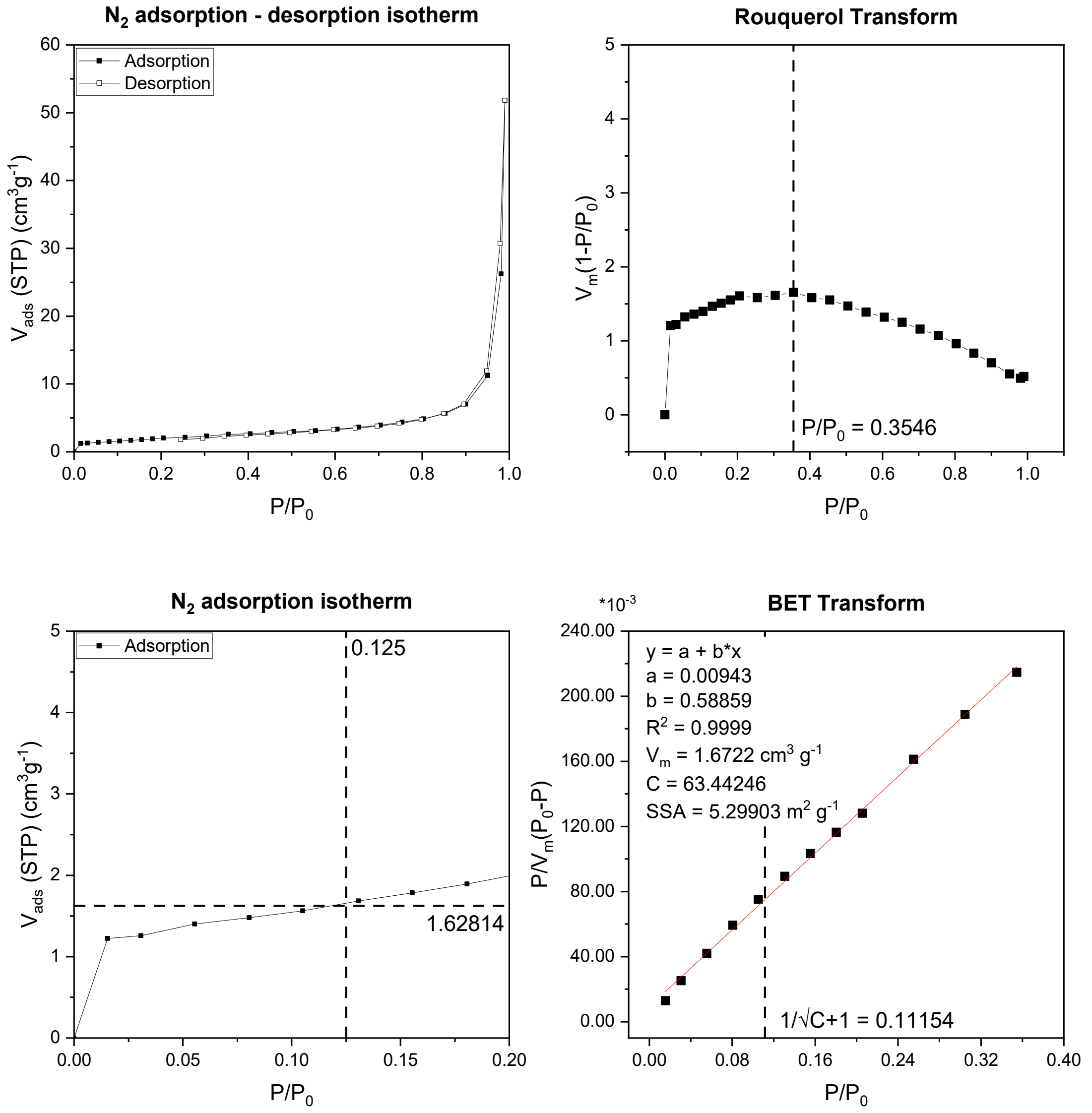

Figure S2. Porosity analysis of CeO_2 cubes. a) N_2 adsorption and desorption isotherms at 77 K; b) Rouquerol transform plot; c) zoom-in of the N_2 adsorption isotherm at 77 K and d) BET transform plot.

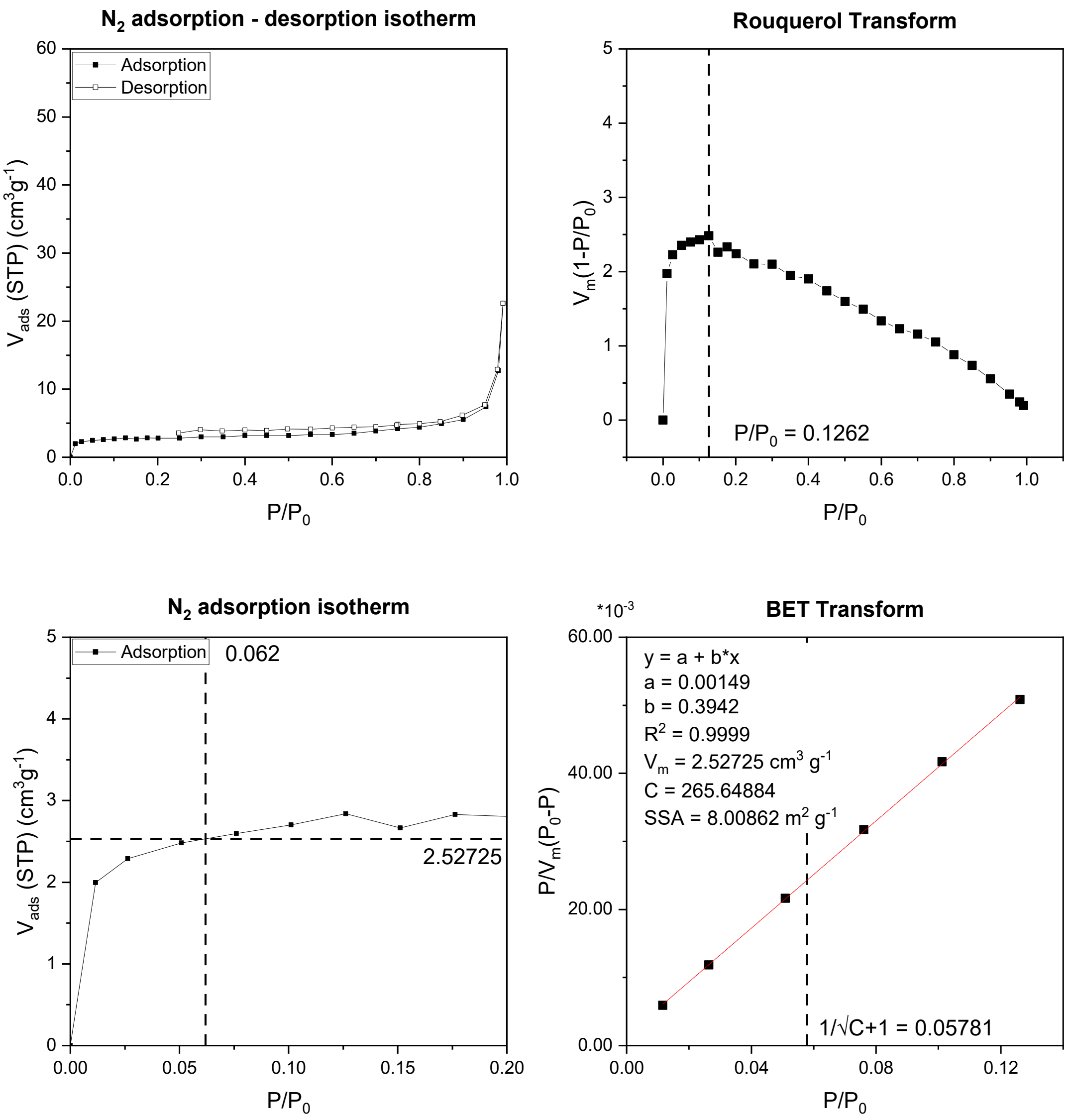

Figure S3. Porosity analysis of CeO_2 octahedra. a) N_2 adsorption and desorption isotherms at 77 K; b) Rouquerol transform plot; c) zoom-in of the N_2 adsorption isotherm at 77 K and d) BET transform plot.

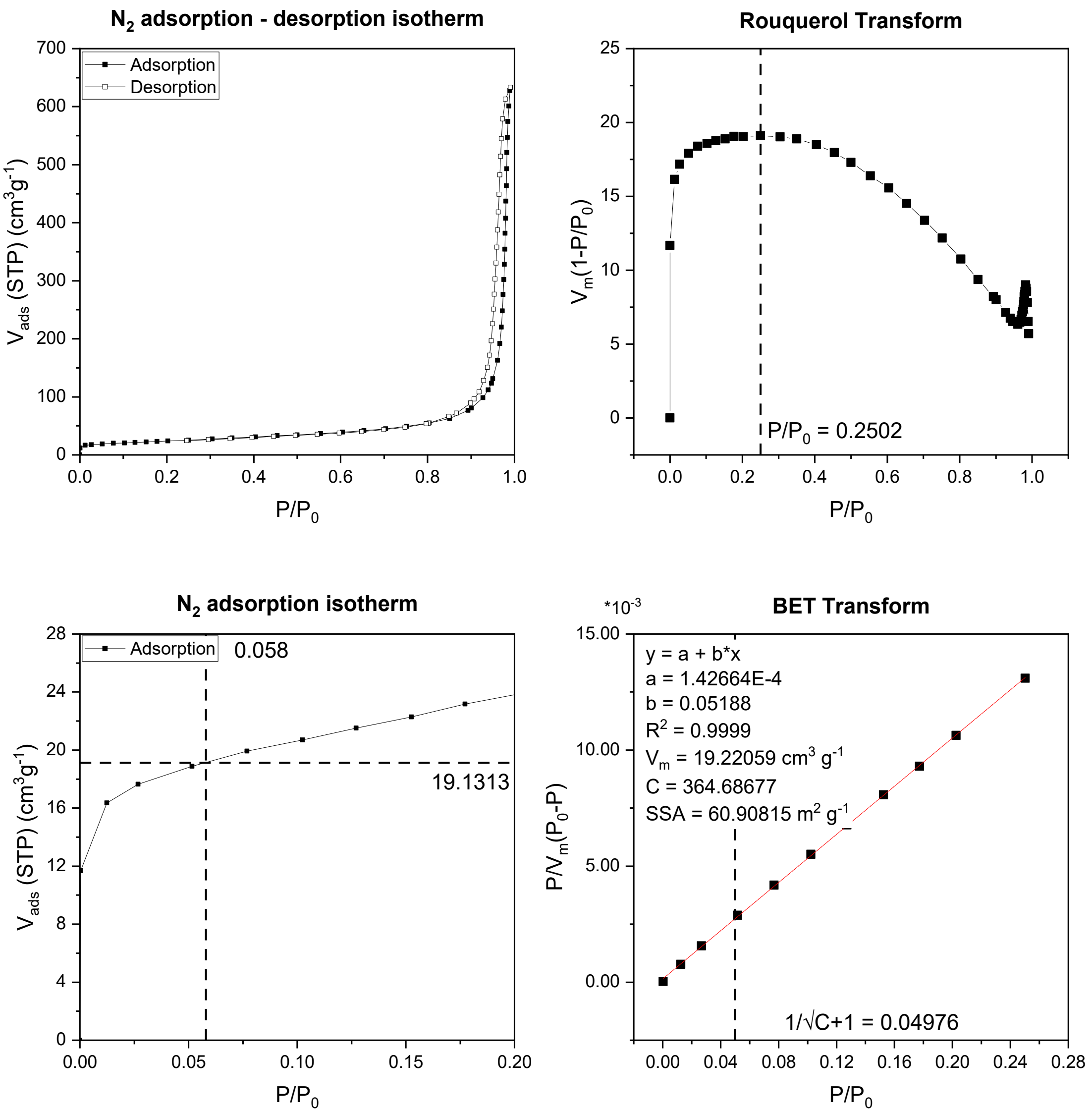

Figure S4. Porosity analysis of CeO_2 rods. a) N_2 adsorption and desorption isotherms at 77 K; b) Rouquerol transform plot; c) zoom-in of the N_2 adsorption isotherm at 77 K and d) BET transform plot.

3. Additional calculations surface content platinum

Table S1. Bulk and surface compositions of Pt_1@cube, Pt_1@octa and Pt_1@rod.

Catalyst	Total Pt content (wt%) [a]	Surface Pt content (at%) [b]	SSA_{BET} (m^2g^{-1}) [c]	Surface Pt content (wt%) [d]	Surface Pt content (μmol g^{-1}) [d]	Average Pt_1-Pt_1 distance (nm) [d]	Pt atom density (atoms per nm^2) [d]
Pt_1@cube	0.08	0.21	5	0.08	4.1	1.4	0.494
Pt_1@octa	0.07	0.20	8	0.07	3.6	1.7	0.270
Pt_1@rod	0.06	0.04	61	0.02	0.8	11.0	0.008

[a] Based on XPS, [b] Based on ICP-OES, [c] Based on N_2 physisorption and [d] assuming that all Pt is present at the surface for cubes because they have the least amount of surface defects based on EPR (**Figure 1f**). Calculations are based on Eq. S1–3.

$$\theta\ (mol\ g^{-1}) = \frac{Pt\ loading\ (wt\%)}{100 * A_r°(Pt, g\ mol^{-1})} \#(S1)$$

$$d(Pt_1 - Pt_1)\ (nm) = \sqrt{\frac{SSA\ (m^2g^{-1}) *\ 10^{18}}{\theta\ (mol\ g^{-1}) * N_A}} \#(S2)$$

$$Pt\ atoms\ per\ nm^2 = \frac{\theta\ (mol\ g^{-1}) * N_A}{SSA\ (m^2g^{-1}) *\ 10^{18}} \#(S3)$$

4. X-ray photoelectron spectroscopy

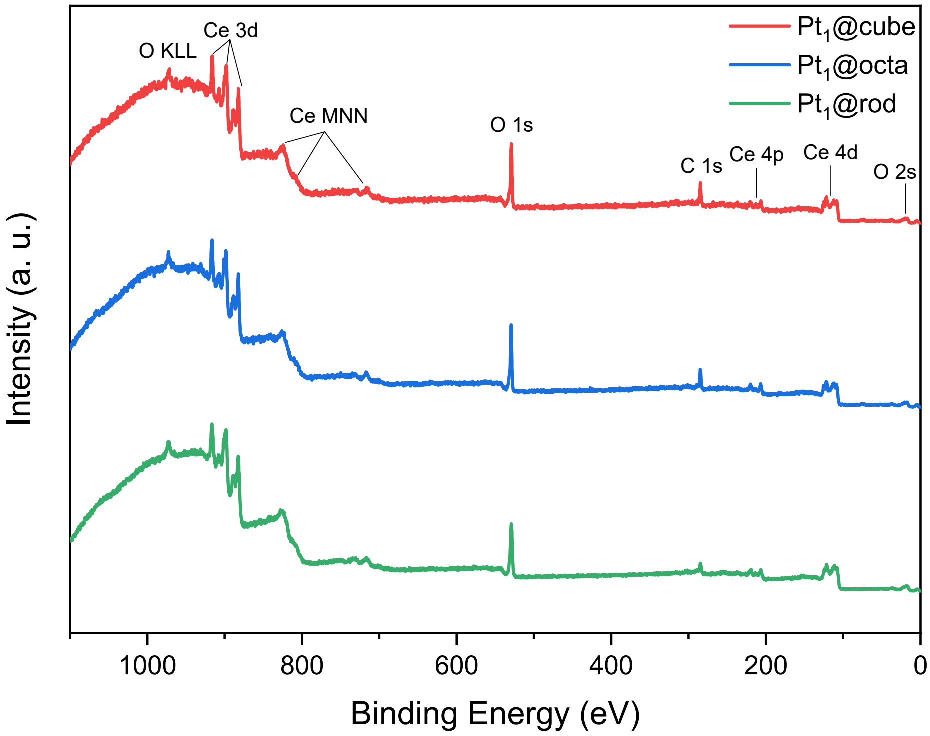

Figure S5. Survey XPS spectra of Pt_1@cube, Pt_1@octa and Pt_1@rod. The peaks for Pt are only observed in detailed measurements owing to their low surface concentrations.

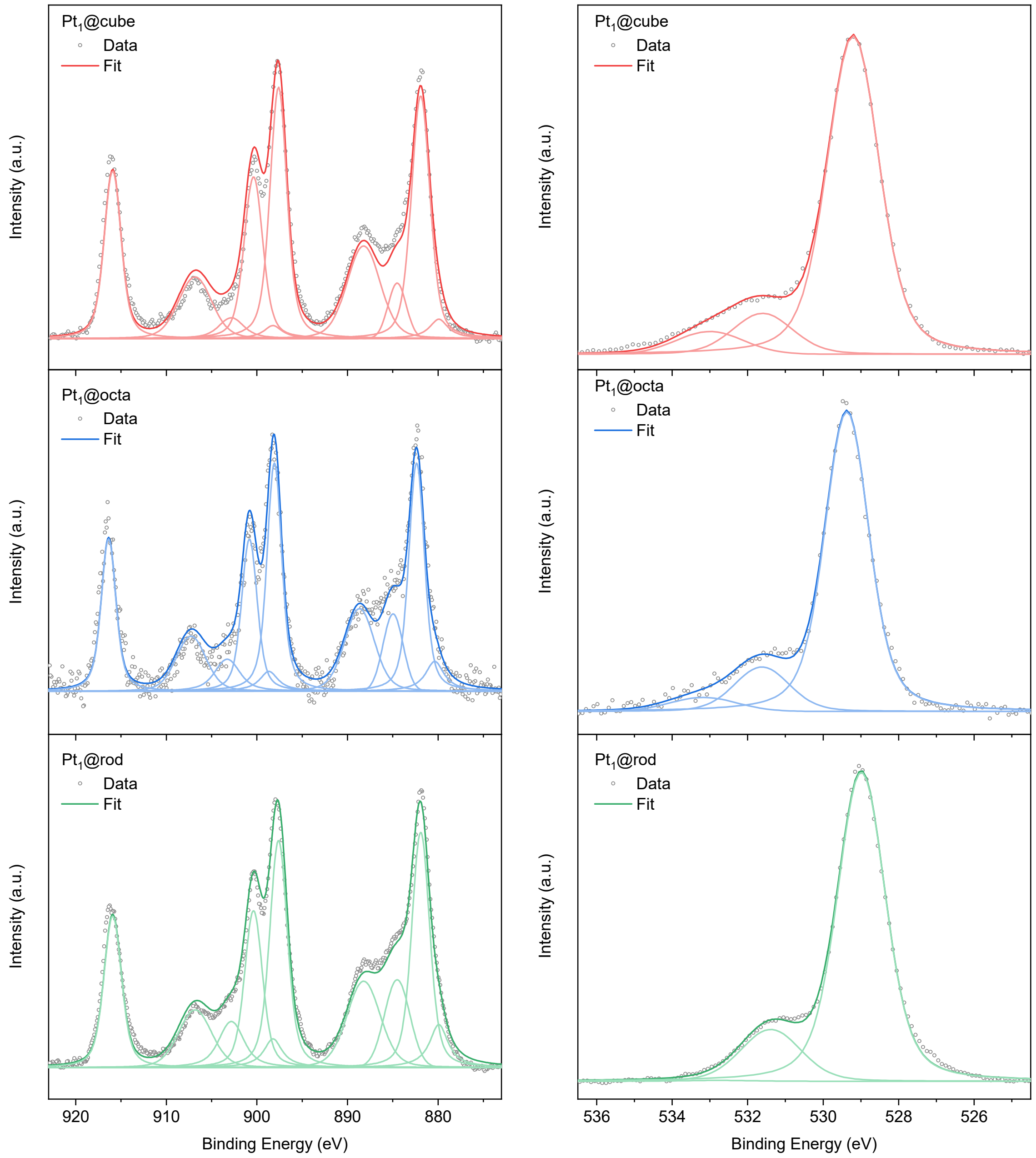

Figure S6. High-resolution XPS spectra of the Ce 3d region (left) and the O 1s region (right) of Pt_1@cube, Pt_1@octa and Pt_1@rod.

Table S2. Surface Ce^{3+} and O_V fractions of Pt_1@cube, Pt_1@octa and Pt_1@rod.

Catalyst	$Ce^{3+}/(Ce^{3+} + Ce^{4+})$ (%)	$O_V/(O_V+O_L)$ (%)
Pt_1@cube	10	12
Pt_1@octa	18	15
Pt_1@rod	21	16

5. X-ray absorption spectroscopy

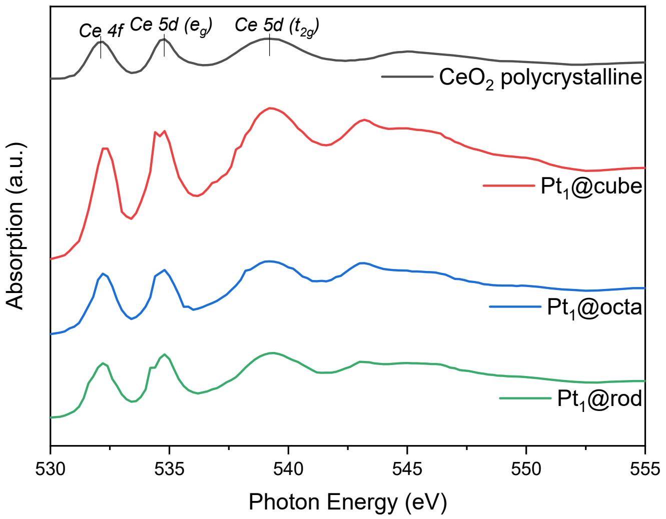

Figure S7. O K-edge XAS of Pt_1@cube, Pt_1@octa and Pt_1@rod compared with polycrystalline CeO_2 as reference.

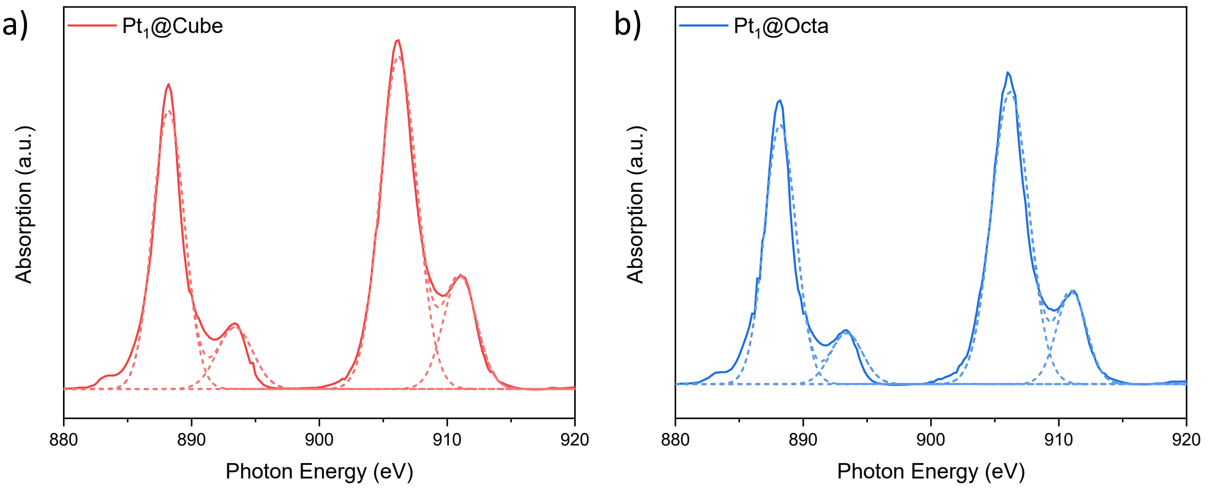

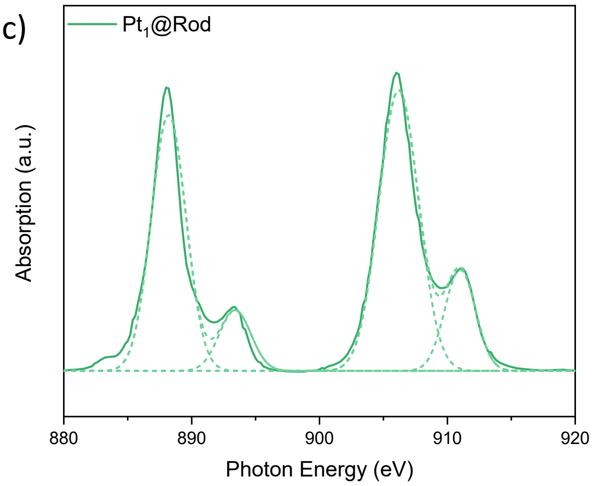

d)

Catalyst	I_{M_5} (888.2 eV)	I_{M_4} (906.2 eV)	I_{M_5}/I_{M_4}
Pt_1@cube	2.93	4.09	0.72
Pt_1@octa	2.32	3.12	0.74
Pt_1@rod	1.90	2.39	0.80

Figure S8. Ce $M_{4,5}$-edge XAS of (a) Pt_1@cube, (b) Pt_1@octa, (c) Pt_1@rod and (d) the integrated M_5 and M_4 areas and their respective ratios.

6. CO diffuse reflectance infrared Fourier transform spectroscopy

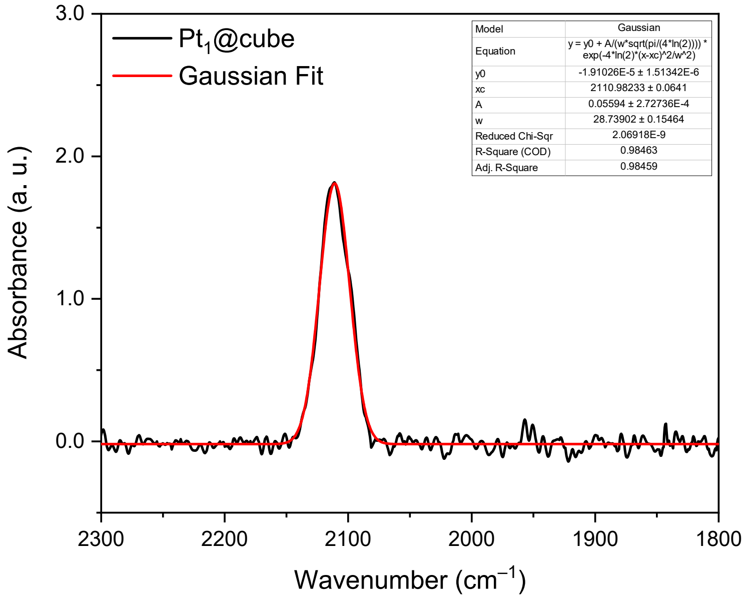

Figure S9. CO diffuse reflectance infrared Fourier-transform spectroscopy (CO-DRIFTS) spectrum of Pt_1@cube and the corresponding Gaussian fit.

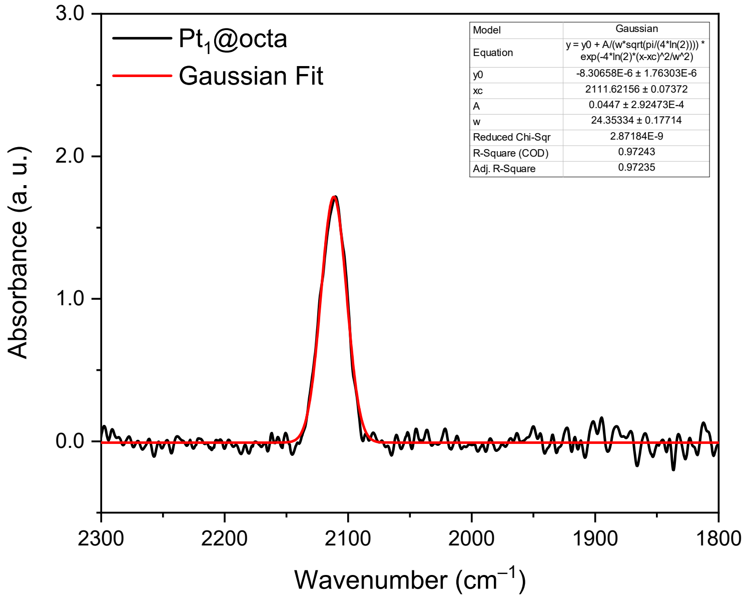

Figure S10. CO diffuse reflectance infrared Fourier-transform spectroscopy (CO-DRIFTS) spectrum of Pt_1@octa and the corresponding Gaussian fit.

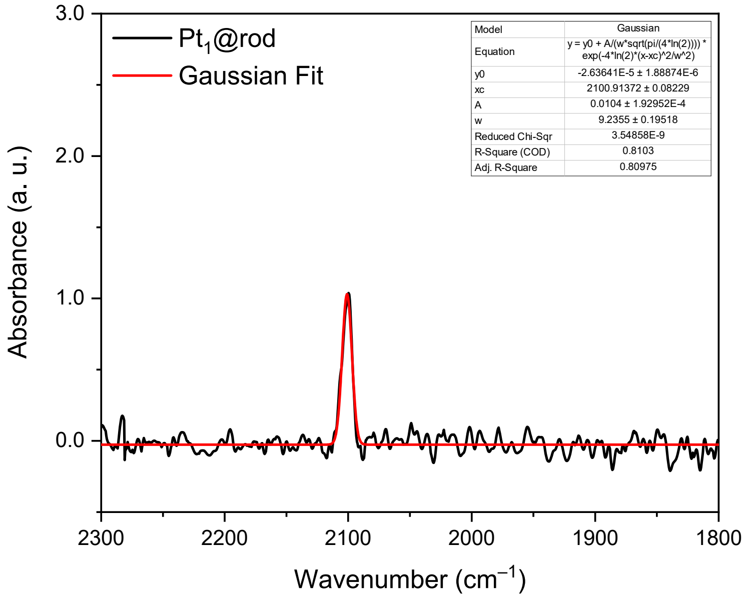

Figure S11. CO diffuse reflectance infrared Fourier-transform spectroscopy (CO-DRIFTS) spectrum of Pt_1@rod and the corresponding Gaussian fit.

7. Kinetic studies of ammonia borane hydrolysis

Table S3. Total conversion during the non-isothermal ammonia borane hydrolysis experiments using Pt_1@cube, Pt_1@octa and Pt_1@rod and the pure supports.

Catalyst	Conversion (%)
Cube	<1
Pt_1@cube	14
Octa	<1
Pt_1@octa	18
Rod	<1
Pt_1@rod	3

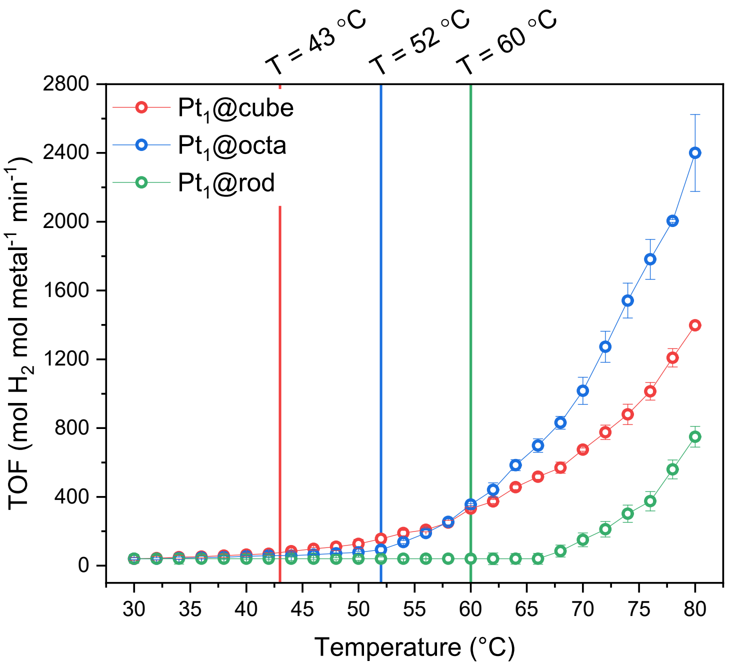

Figure S12. Turnover frequency (TOF) at various temperatures for the hydrolysis of ammonia borane catalyzed by Pt_1@cube, Pt_1@octa and Pt_1@rod. Each data point represents a window average of 10–50 measurements using equal time interpolation and all experiments were performed in duplo. The data shown here indicates the averaged values and the error bars indicate a 95% confidence interval. The vertical lines indicate the temperatures at which the corresponding Arrhenius plot show a kink (**Figure 3**).

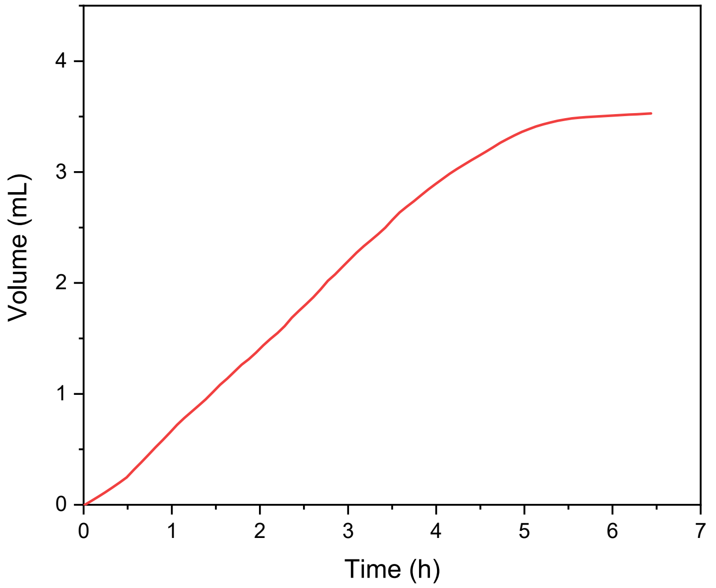

Figure S13. Kinetic profile of Pt_1@cube catalyzed ammonia borane hydrolysis at 40 °C based on *ca*. 400 data points.

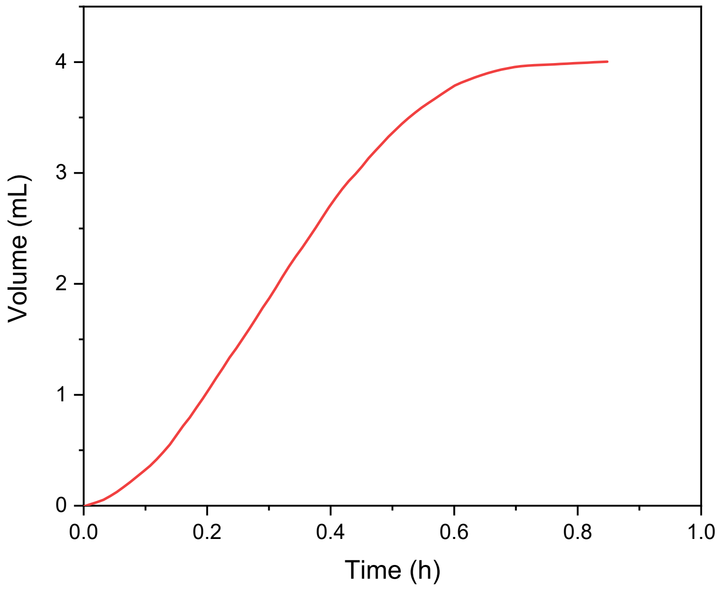

Figure S14. Kinetic profile of Pt_1@cube catalyzed ammonia borane hydrolysis at 65 °C based on ca. 400 data points.

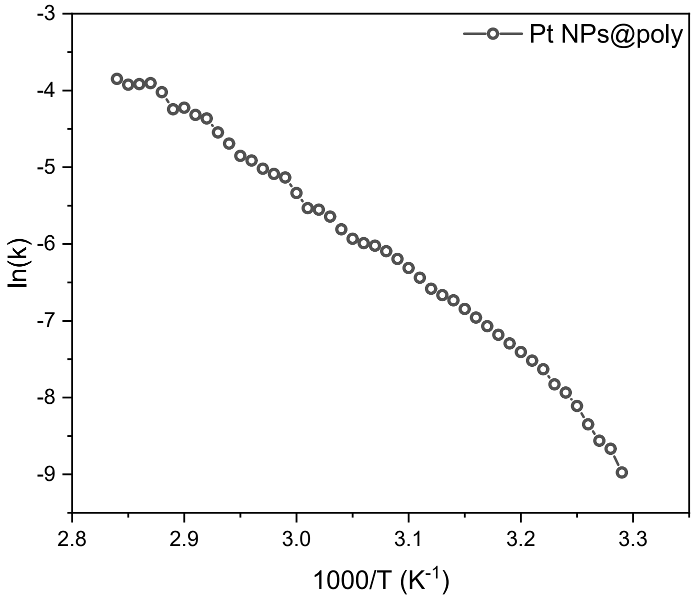

Figure S15. Arrhenius plot of platinum nanoparticles supported on polycrystalline cerium dioxide in the hydrolysis of ammonia borane. Each data point represents a window average of 30 individual measurements.

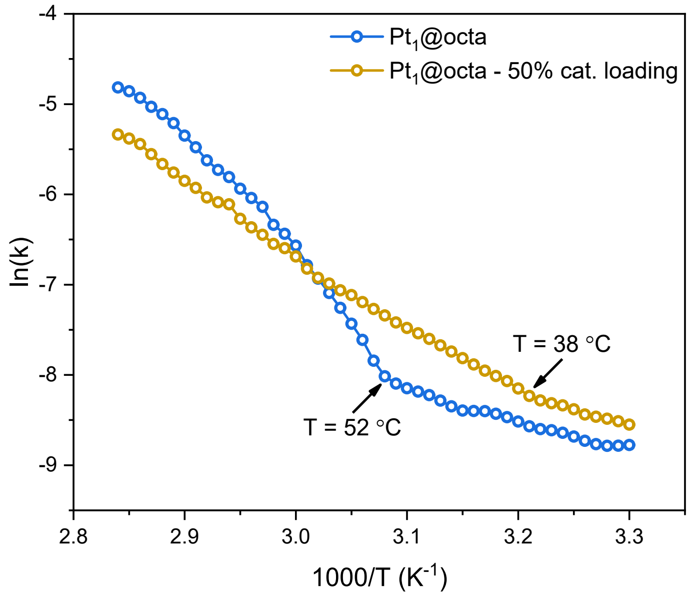

Figure S16. Arrhenius plots of Pt_1@octa and Pt_1@octa at 50% catalyst loading in the hydrolysis of ammonia borane. Each data point represents a window average of 30 individual measurements. All experiments were performed in duplo and the data shown here represent the averaged values.

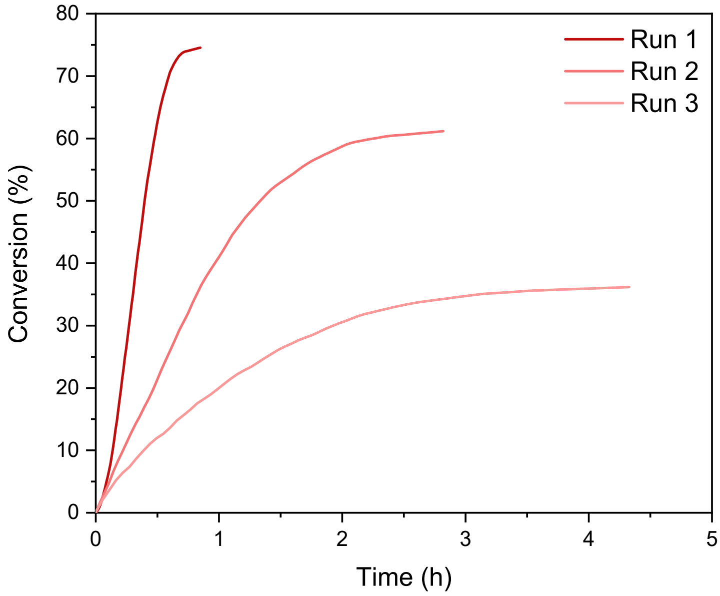

Figure S17. Kinetic profiles of Pt_1@cube catalysed ammonia borane hydrolysis for three consecutive runs.

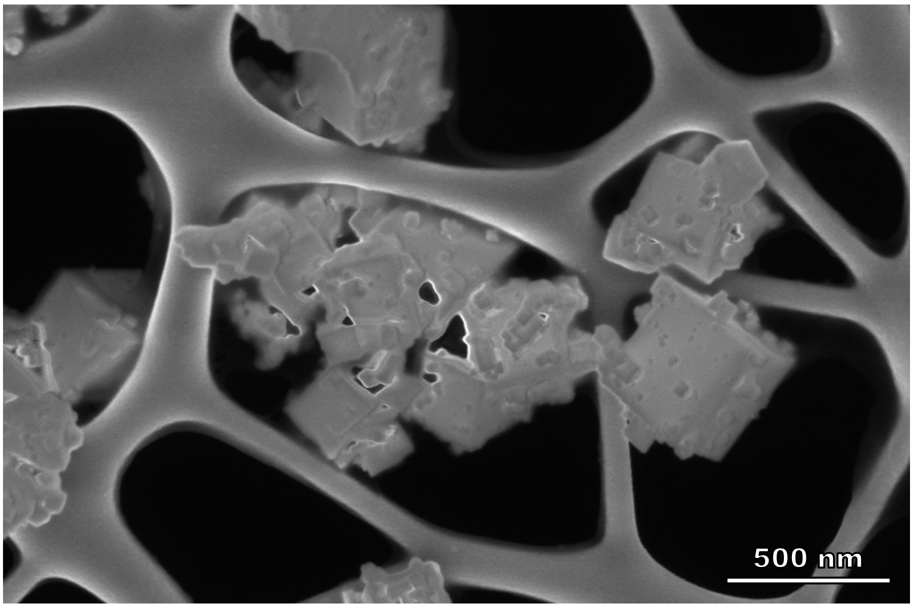

Figure S18. Scanning electron microscope (SEM) images of spent Pt_1@cube showing deposition of the metaborate salts, which is absent before the reaction (*cf.* **Figure S1**).
